# Cortical myelin mapping in antipsychotic medication-naïve, first-episode psychosis patients

**DOI:** 10.1038/s41386-025-02137-9

**Published:** 2025-05-23

**Authors:** Victoria L. King, Gerhard Hellemann, Adrienne C. Lahti, Matthew Defenderfer, Jill R. Glausier, Hui Zhang, Nina V. Kraguljac

**Affiliations:** 1 Department of Psychology, University of Alabama at Birmingham, Birmingham, AL USA; 2 Department of Psychiatry and Behavioral Neurobiology, University of Alabama at Birmingham, Birmingham, AL USA; 3 Department of Psychiatry and Behavioral Health, The Ohio State University, Columbus, OH USA; 4 Department of Biostatistics, University of Alabama at Birmingham, Birmingham, AL USA; 5 Research Computing, Information Technology, University of Alabama at Birmingham, Birmingham, AL USA; 6 Department of Psychiatry, University of Pittsburgh, Pittsburgh, PA USA; 7 Centre for Medical Image Computing, Department of Computer Science, University College London, London, UK

**Keywords:** Schizophrenia, Biomarkers, Schizophrenia, Translational research

## Abstract

While white matter myelin primarily functions to accelerate conduction velocity and has been extensively studied in schizophrenia-spectrum disorders (SSD), less is known about the role of gray matter myelin in SSD. Cortical myelination occurs mostly on the proximal axons of parvalbumin positive (PV+) interneurons, where it assists in trophic support and experience-dependent plasticity. Given the role of PV+ interneuron dysfunction in SSD, it is critical to advance our understanding of cortical myelin pathology in this context. Here, we quantified myelin maps using the T1w/T2w ratio in a large group of antipsychotic medication-naïve, first-episode psychosis patients. We compared myelin content between patients (*N* = 91) and controls (*N* = 107) using a MANCOVA and calculated zero-order correlations with the discriminant function for each region, then used a machine learning approach to identify the most parsimonious constellation of cortical regions driving group differences using a stepwise algorithm. Group membership was significantly associated with T1w/T2w ratio (Wilks Lambda = 0.09, *p* < 0.01), where patients had higher myelin values compared to healthy controls. We identified a subset of 16 regions, primarily located in association cortices, that were sufficient to explain group differences. Here, we report an increase in the cortical T1w/T2w ratio in association cortices in first-episode psychosis. We suggest that faulty myelin compaction during this critical developmental period could contribute to PV+ interneuron pathology and cortical microcircuit disruptions resulting in the clinical phenotype. With additional empirical support from future studies, novel treatment strategies targeting cortical myelin could have potential to mitigate circuit dysfunction in the illness.

## Introduction

Myelin is most well-known to act as an insulating factor for long-range white matter axons, which speeds up the conduction velocity of action potentials in order to transmit signals to gray matter brain regions. Seminal studies indicate that oligodendrocytes, which form myelin sheaths, are generated early in life, and they continue to myelinate axons into early adulthood until the axon surface is compactly wrapped [1]. Disruptions in white matter tract myelination can affect the function of cortical neural circuits that support cognition, motivation, and emotion. Several genetic [2–4], postmortem morphologic [5–8], and in vivo neuroimaging studies of white matter and oligodendrocytes [9–11] support the idea that cortical dysmyelination is a critical pathophysiological mechanism underlying aberrant signal processing, a core feature of schizophrenia-spectrum disorders (SSD). Given that myelination and symptom onset in schizophrenia tend to peak around the same time [12, 13], it is hypothesized that myelin-related dysfunction could explain the long latency between the onset of neuropathological changes and the clinical onset of the disorder [14].

In contrast to white matter findings, far less well-known is the role of myelin in gray matter. Grey matter myelin development occurs in a caudal to rostral direction, such that primary sensory and motor areas are myelinated before association and limbic cortices; this spatial variation defines the sensorimotor-association (S-A) axis [15]. Interestingly, many patterns of neurodevelopment occur along this axis, including cortical thickness, functional connectivity patterns, and cellular microstructure [15]. Previous in vivo imaging studies of cortical gray matter myelin in individuals with SSD report reduced cortical myelin in sensorimotor [16, 17] and association cortices [18, 19], although interpretation of these studies is confounded by sample sizes and medication status. More recently, however, Wei and colleagues have identified depth-dependent patterns of myelination in a group of antipsychotic medication-naïve patients [20], where there was a reduction of myelin in the posterior central gyrus and insular gyrus in the middle cortical layer, but an increase in the superficial layer of the supramarginal gyrus and in the superficial and middle layer of the inferior parietal lobule (primarily association areas). Taken together, previous in vivo myelin mapping studies suggest abnormal myelination in SSD are differentially expressed across the S-A axis.

While myelin is less abundant in cortical gray matter than in white matter, cortical neurons exhibit diverse, discontinuous myelination patterns that are influenced by cell type and axonal diameter [21]. This pattern of interspersed myelin segments, found on both pyramidal neuron and interneuron axons, points to their potential functionality; myelin internodes proximally located on the axon are likely to ensure propagation of action potentials, while more distally located myelin may be an important factor in the inhibition of synapse formation [22]. Specifically, pyramidal neurons display layer-dependent differences in myelination, where this pattern of intermittent myelination is particularly exhibited in layer II/III [23].

Glutamatergic signaling from pyramidal neurons is controlled by GABAergic interneurons, particularly those expressing parvalbumin (PV), a calcium-binding protein that allows for their fast-spiking activity [22, 24]. Stedehouder and colleagues [22] discovered that gray matter myelin is preferentially expressed on PV-containing (PV+) interneurons in layer II/III, and that PV+ interneuron myelination accounts for a substantial proportion of cortical myelin. Interestingly, expression of PV+ interneurons decreases along the S-A axis such that sensorimotor cortices display a higher density and association cortices display a lower density [15]. Due to their fast-spiking behavior, PV+ interneurons play a large role in regulating excitatory signaling in pyramidal neurons [25]. Thus, abnormalities in PV+ neuron myelination can lead to disruptions in the excitatory/inhibitory balance and failure to coordinate signaling between cortical brain regions; two hallmark features of schizophrenia [25].

In the present study, we enrolled a large group of racially-diverse, antipsychotic medication-naïve, first-episode psychosis patients (*N* = 91) and healthy controls (*N* = 107) matched on key demographic variables to assess cortical gray matter myelin pathology in vivo using the T1/T2 ratio as a proxy of cortical myelin content [26]. Here, we moved away from mass univariate analyses, and instead performed canonical discriminant analyses which account for disease effects in multiple brain regions at the same time. Using post hoc analyses, we then investigated relationships between myelination patterns and a number of clinically relevant variables, including duration of untreated psychosis (DUP), clinical symptom severity and cognition in the patient group.

## Patients and methods

### Participants and study design

We recruited first-episode patients (FEP) from outpatient, inpatient, and emergency room settings at The University of Alabama at Birmingham (UAB). After being deemed to have capacity to give consent [27], written informed consent (and informed assent for participants <18 years old) was obtained and the patients were enrolled. We also recruited healthy controls matched on age, sex, and socioeconomic status (SES). The study was approved by the UAB Institutional Review Board.

We excluded participants if they had one or more of the following: major neurological conditions, history of head trauma with loss of consciousness, substance use disorders (excluding nicotine and cannabis) within one month of imaging, were pregnant or breastfeeding, or had MRI contraindications. Patients were either medication-naïve or had no more than five days of lifetime antipsychotic exposure prior to enrollment. Controls with a personal history of a mental illness or family history in a first-degree relative of a psychotic disorder were also excluded.

Diagnoses were established in consensus by two board certified psychiatrists (ACL and NVK) using the Mini-International Neuropsychiatric Interview (MINI) or the Diagnostic Interview for Genetic Studies (DIGS), along with medical records and reports from families as available. We included patients with a final diagnosis of schizophrenia, schizoaffective disorder, schizophreniform disorder, brief psychotic disorder, and psychosis not otherwise specified (NOS). We operationally defined DUP as the time between first onset of positive symptoms and the start of treatment. The time of symptom onset was determined in consensus via clinical interviews with the patient and family members as well as medical records and clinical observations and assessments over several months of follow up. We assessed symptom severity and cognition with the Brief Psychiatric Rating Scale (BPRS) and the Repeatable Battery for the Assessment of Neuropsychological Status (RBANS).

### Demographics

We recruited a total of 213 subjects. Of those subjects, 108 were healthy controls and 105 were FEP. For complete demographics, see Table 1. We excluded 1 control and 7 patients due to poor quality scans or missing participant information, and 7 patients due to a diagnosis of a mood disorder (major depressive disorder, bipolar disorder). There were no group differences in exclusions for signal loss or presence of artifact, as we only excluded one healthy control and two patients due to these factors. Final statistical analyses included 198 subjects (107 controls, 91 patients).

**Table 1:** Demographics and clinical measures.

	Healthy controls (*n* = 108)	First-episode psychosis (*n* = 105)	F/t/χ^2^	*p* value
**Demographics**				
Gender (%male)	54.60	61.90	1.15	0.28
Age	23.88 (5.27)	23.39 (5.60)	0.43	0.51
Socioeconomic status^a^	4.42 (3.92)	5.56 (4.78)	3.62	0.06
Duration of untreated psychosis (months)		23.52 (40.48)		
**Diagnosis**				
Schizophrenia		52		
Schizoaffective disorder		18		
Schizophreniform disorder		6		
Psychosis NOS		17		
Brief psychotic disorder		5		
**Clinical measures**				
BPRS^b^				
Total score		47.73 (11.50)		
Positive symptom score		14.96 (3.95)		
Negative symptom score		5.38 (2.95)		
RBANS^c^				
Total score	93.45 (10.22)	76.00 (16.01)	81.83	< 0.001

All numbers reported indicate mean (standard deviation) unless noted otherwise.

^a^Ranks determined from Diagnostic Interview for Genetic Studies (1–18 scale); higher rank (lower numerical value) corresponds to higher socioeconomic status.

^b^Brief Psychiatric Rating Scale (1–7 scale) positive score contains the items conceptual disorganization, hallucinatory behavior, unusual thought content and suspiciousness; negative score contains the items (emotional withdrawal, motor retardation, and blunted affect).

^c^Repeatable Battery for the Assessment of Neuropsychological Status.

### Data acquisition

All imaging was performed on a 3 T Siemens MAGNETOM Prisma scanner (Erlangen, Germany) equipped with a 20-channel head coil. T1w images were acquired using a T1-weighted magnetization prepared rapid acquisition gradient-echo (MPRAGE) scan [TR/TE/inversion time: 2400/2.22/1000 ms; flip angle: 8°; echo spacing: 7.5 ms; GRAPPA factor 2; 256 × 256 × 208 matrix; 0.8 × 0.8 × 0.8 mm^3^ voxels]. T2w images were acquired using a T2-weighted sampling perfection with application optimized contrast using different angle evolutions (SPACE) [TR/TE: 3200/56; flip angle: variable; echo spacing: 3.52 ms; GRAPPA factor: 2; 256 × 256 × 208 matrix, 0.8 × 0.8 × 0.8 mm^3^ voxels].

### T1w/T2w myelin map processing and quality control

T1w and T2w data were processed using the minimal HCP preprocessing pipelines [28]. Bias field correction and creation of the T1w/T2w ratio maps was performed as described in [26]. We verified protocol adherence and visually inspected all raw T1 and T2 images for signal loss and presence of artifact. We then confirmed that skull stripping and acpc alignment was successful. This was followed by visual inspection of individual myelin maps to check for anomalies using myelin maps described by Glasser and colleagues [28] as a reference.

### Statistical analyses

We ran a region-of-interest (ROI) analysis using Connectome Workbench (Version 1.5.0, https://github.com/Washington-University/workbench). We used the Destrieux atlas [29] to parcellate each subjects’ myelin map and computed the average myelin value for each of the 148 regions of interest (ROIs).

To determine if there were multivariate differences between patients and controls, we used a multivariate analysis of covariance (MANCOVA). This technique is closely related to canonical correlation analysis, which has been used successfully in imaging data [30, 31]. It projects observed data onto a vector that has the highest correlation with the vector defined by the grouping variables and explains the most variance in the data (first canonical discriminant). In addition to determining which linear combination is most strongly associated with group membership, it provides statistical tests to determine if this association is statistically significant or spurious. We conducted post hoc analyses when the omnibus test was significant to determine the pattern of group differences. All analyses were conducted on age- and sex- corrected and z-scored values to ensure variance homogeneity and minimize confounding.

To further explore the results, we calculated zero-order correlations with the discriminant function for each region. We choose to present the zero-order correlations/ structure matrix instead of the raw discriminant function because it allows for the direct comparison between different regions independent of the correlation between the different outcomes. In addition, we also used a machine learning approach to identify the smallest subset of outcomes that can be used to define a canonical linear discriminant.

In an exploratory fashion, we ran a linear regression analysis to determine whether myelination in the 16 regions significantly predicted duration of untreated psychosis (DUP), RBANS scores, or BPRS scores, after controlling for age, sex, and socioeconomic status (SES). We also performed correlations with cortical thickness to investigate whether our findings were dependent on this measure.

## Results

### T1w/T2w ratio analysis

**Fig. 1:**
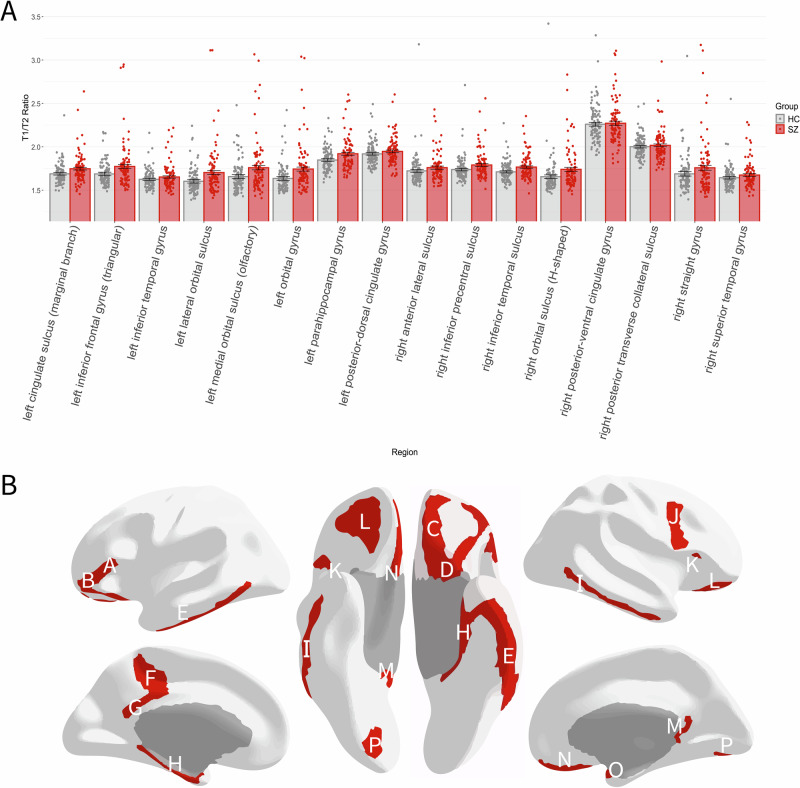
Regions which display increased myelin in FEP versus controls. Panel (**A**) shows the results of the ANOVA, revealing 16 regions which are sufficient to explain group differences of myelination between healthy controls and first-episode patients. Panel (**B**) depicts these regions on a brain surface; A left inferior frontal gyrus, B left lateral orbital sulcus, C left medial orbital sulcus, D left orbital gyrus, E left inferior temporal gyrus, F left cingulate sulcus, G left posterior-dorsal cingulate gyrus, H left parahippocampal gyrus, I right inferior temporal sulcus, J right inferior precentral sulcus, K right anterior lateral sulcus, L right H-shaped orbital sulcus, M right posterior-ventral cingulate gyrus, N right straight gyrus, O right planum polare, P right posterior collateral sulcus.

When determining if group membership (controls vs. patients) was associated with myelination across all ROIs simultaneously, we found a significant signal (Wilks Lambda = 0.09, *p* < 0.01). Using a stepwise algorithm to determine the most parsimonious subset of the data that contains this signal yielded a smaller subset of 16 regions which is sufficient to describe this relationship (Fig. 1, Wilks lambda = 0.55, *p* < 0.01). The correlation of the canonical variate based on all ROIs and the canonical variate based on only these 16 ROIs is r = 0.74. All 16 regions showed increased T1w/T2w ratio in patients compared to controls and were primarily multimodal association areas.

### Post hoc analyses

After correcting for multiple comparisons using Bonferroni correction, there were no correlations between myelin values and DUP, RBANS, or BPRS. There were no group differences in cortical thickness in any of the 16 regions identified in the main analysis when controlling for age, sex, and SES. In addition, after correction for multiple comparisons, only two regions showed significant correlations between T1w/T2w ratio values and cortical thickness in patients (left orbital gyrus (r = −0.434, *p* < 0.001) and right straight gyrus (r = −0.402, *p* < 0.001).

## Discussion

In an effort to better understand cortical myelin pathology in schizophrenia-spectrum disorders, we quantified cortical myelination in vivo in a large group of antipsychotic medication-naïve first-episode psychosis patients and observed that aberrant cortical myelination was already detectable. In addition, we found that the subset of brain regions that was sufficient to describe group differences largely spanned across multimodal association regions. We did not, however, identify significant associations between clinical variables and myelin content in those regions. Together, our findings imply that myelin pathology may be a central feature of the illness rather than a byproduct of illness chronicity or antipsychotic medication effects.

Interestingly, we found an increase in the cortical T1w/T2w ratio, while most previous myelin mapping studies have observed decreased cortical myelin [16–19]. The only other study in medication-naïve, first-episode psychosis patients also discovered an increase in apparent myelin content in multiple cortical regions [20], though they also noted regions with decreased cortical myelin. It is possible that improper myelin compaction could be underlying this increase. One of the factors contributing to this ratio is the amount of free water and myelin bound water [26]. With less compaction, more myelin-bound water would be trapped between the myelin sheaths, which in turn, could result in an increase of the T1w/T2w ratio.

Myelin compaction refers to the process by which the multiple layers of oligodendrocyte-derived membrane wrap around the axon to form the compact myelin sheath. This process is crucial for efficient action potential conduction and neural circuit stability. Several myelin-related proteins are involved in this process. For instance, myelin basic protein (MBP) and proteolipid protein (PLP) play a central role by promoting the adhesion of the myelin lamellae, facilitating the compaction of the myelin sheath [32]. In contrast, 2’,3’-cyclic nucleotide 3’-phosphodiesterase (CNP) and myelin-associated glycoprotein (MAG) prevent excessive compaction, maintaining synaptic plasticity and ensuring that appropriate connections are made—especially during the adolescent period [32]. A precise balance of these proteins is essential for healthy myelination. Previous literature has shown that dysregulation of these proteins (under- or overexpression) is evident in schizophrenia [33].

Several lines of evidence support the idea that myelin compaction may be faulty in schizophrenia. Myelin-associated proteins, including myelin basic protein (MBP) and proteolipid protein (PLP) [32] act in conjunction as a ‘plasticity brake’ [34] by compacting myelin through increasing the adhesiveness of the membrane [35]. A decrease in MBP and PLP in schizophrenia has been reported in several gene expression and proteome studies (reviewed in [36]). Genetic deletion of MBP in preclinical models results in the presence of non-compacted myelin around axons of parvalbumin positive interneurons [37]. In addition, a postmortem study in schizophrenia demonstrated a loss of compactness of myelin lamellae in myelinated fibers in the frontal cortex [38]. Together, these data support our interpretation of an increased T1w/T2w ratio as a reflection of insufficient myelin compaction.

There are two different possible interpretations of an increase in the T1w/T2w ratio; (1) if cortical thickness is constant, an increase could be the result of increases in myelinated axons; (2) if cortical thickness is decreased, the apparent increase in myelin could be due to a decrease in non-myelinated dendrites resulting in a relative increase in myelin density in the remaining cortex [39]. However, given that cortical thickness itself can be affected by cortical myelin content, it is difficult to ascertain specificity. While our findings revealed two regions which showed a correlation between T1w/T2w ratio and cortical thickness in patients, our overall findings of increased T1w/T2w ratio cannot be attributed to differences in cortical thickness. Future studies combining the T1w/T2w ratio with other modalities such as g-ratio imaging, where cortical myelin water fraction and neurite density are measured, would allow more specific interpretations as to the pathological mechanism underlying the apparent increase in myelin we observed here.

Finally, we did not observe significant associations between cortical myelin and clinical variables such as BPRS or RBANS scores. This is consistent with multiple recent studies that also failed to detect robust relationships between cortical myelin and symptom severity after correcting for multiple comparisons [17, 40, 41]. Wei and colleagues [20] found that years of education moderated these associations, highlighting the potential influence of demographic factors. While another study reported some significant relationships between intracortical myelin and PANSS scores [42], methodological differences limit direct comparability to our work. Together, these findings suggest that cortical myelin may reflect more stable, trait-like neurobiological differences rather than acute symptom fluctuations.

As discussed, PV+ interneuron dysfunction has been proposed as a fundamental mechanism underlying schizophrenia, where GABAergic signaling is reduced and in turn results in decreased inhibition of glutamatergic pyramidal cells and brain circuit dysfunction [43]. This idea is supported by converging lines of experimental data from animal models [44–46], pharmacological challenge studies with ketamine [47], and in vivo neuroimaging data [48–52]. Interestingly, several postmortem studies found that neither the number of axons nor axonal morphology of PV+ interneurons is altered in schizophrenia but rather suggest that parvalbumin protein levels are downregulated [43, 53]. Given the tight interdependence between PV+ interneurons and their myelin sheaths, it is suggested that PV+ interneuron dysfunction and impaired myelin pathology may converge in schizophrenia [54]. However, it is not known if either of these cell types drives pathology of the other, or if it is a faulty interaction between them that causes a reduction in parvalbumin and myelin dysfunction.

One potential point of convergence between interneuron dysfunction, myelination abnormalities, and schizophrenia risk involves the NRG1–ErbB4 signaling pathway. This pathway has been strongly associated with schizophrenia susceptibility [55] and plays a broad role in neural development, including neuronal migration, synaptogenesis, and oligodendrocyte specification, differentiation, and survival [56, 57]. Although it is not currently known whether any schizophrenia-associated genes are specifically linked to the myelination of GABAergic interneurons, NRG1–ErbB4 signaling represents a compelling candidate. NRG1 dysregulation, whether through increased or decreased expression, has been shown to alter synaptic plasticity and disrupt cortical excitatory/inhibitory balance, potentially by influencing dendritic spine growth and interneuron activity [56]. Importantly, ErbB4 expression is enriched in parvalbumin-expressing (PV+) interneurons, and selective loss of ErbB4 in these cells impairs GABAergic circuitry and reduces spine density [58]. While this pathway was not directly assessed in the current study, it may represent a biologically plausible mechanism linking schizophrenia risk genes with both interneuron dysfunction and myelin abnormalities observed in the disorder.

On a more macroscopic level, we found that the regions which provided the most parsimonious solution for explaining group differences in the T1w/T2w ratio were mostly located in association regions of the brain which is consistent with Wei and colleagues who also reported an increase in the ratio in association cortices [20]. Cortical myelin increases substantially in the first decade of life, with a second prominent wave of myelination around 18–20 years of age [59]. This second wave is a uniquely human feature, and predominantly driven by late myelination of association cortices [15]. Here, myelin functions as a structural plasticity brake to ensure the stability of newly formed connections [60] resulting in the closure of this second “critical developmental period” [34]. It is tempting to speculate that dysfunctional myelination during this second wave, which coincides with the typical age of onset in schizophrenia, could constitute the ‘second hit’ in the development of psychosis spectrum disorders.

### Strengths and limitations

One of the critical strengths is that we examined myelin content in vivo in a large group of antipsychotic medication-naïve, first-episode psychosis patients, which is a notoriously difficult to recruit population, but offers a unique perspective of the underlying pathophysiology by mitigating the confounds of antipsychotic medication effects and illness chronicity. It is important that some limitations should also be acknowledged. We chose to quantify myelin using the T1w/T2w ratio to investigate cortical myelin, which is one of the most common myelin mapping methods. It has been suggested that iron could confound the specificity of the T1w/T2w ratio [61], but iron has been shown to be colocalized with myelin [62]. While this is a known limitation, other myelin mapping methods have similar weaknesses. For example, the magnetization transfer ratio (MTR) is sensitive to all macromolecular pools, and therefore not specific to myelin only [63]. We also chose not to do bias field corrections, as it has been shown that it removes both artifactual and genuine cross-participant differences in the T1w/T2w ratio [39]. We instead used on-scanner corrections where a fixed sharply varying receive field from the head coil is removed from the images by the scanner (Siemens PreScan Normalize), which is recommended as a strategy to mitigate bias [39]. Lastly, here we chose to perform a canonical correlation analysis. While the ideal approach may have been to perform a k-fold model with an independent test dataset to maximize independence/reduce overlap between the training sets and potential variability of the fitted model, the sample size of the present study is not sufficient for this approach.

## Conclusion

To conclude, we report an increase in the cortical T1w/T2w ratio in association cortices, where myelination takes place largely between the ages of 13–20, around the same time psychosis spectrum disorders first manifest clinically. We suggest that faulty myelin compaction during this critical developmental period could contribute to PV+ interneuron pathology and cortical microcircuit disruptions resulting in the clinical phenotype. If future studies provide additional empirical support for this, novel treatment strategies targeting cortical myelin could have potential to mitigate circuit dysfunction in the illness.

## Data Availability

The datasets analyzed during the current study are available from the corresponding author on reasonable request.
